# ‘We are the engine’: a focus group study on clinical practice guideline development with European patient advocates for rare congenital malformations and/or intellectual disability

**DOI:** 10.1186/s13023-025-03673-9

**Published:** 2025-04-10

**Authors:** Mirthe Jasmijn Klein Haneveld, Chloé Aymée de Mortier, Anne Hugon, Martina Cornelia Cornel, Charlotte Maria Wilhelmina Gaasterland, Agnies Marguerite van Eeghen

**Affiliations:** 1 Amsterdam UMC, University of Amsterdam, Emma Children’s Hospital, Meibergdreef 9, Amsterdam, The Netherlands; 2 European Reference Network on Rare Congenital Malformations and Rare Intellectual Disability ERN-ITHACA, Clinical Genetics Department, Robert Debré University Hospital, 48 Boulevard Serurier, 75935 Paris, France; 3 Amsterdam Reproduction and Development Research Institute, Amsterdam, The Netherlands; 4 Amsterdam Public Health Research Institute, Amsterdam, The Netherlands; 5 Knowledge Institute of the Dutch Association of Medical Specialists, Mercatorlaan 1200, 3528 BL Utrecht, The Netherlands; 6 Department of Health Services Research, Care and Public Health Research Institute (CAPHRI), Faculty of Health Medicine and Life Sciences (FHML), Maastricht University, Minderbroedersberg 4-6, Maastricht, The Netherlands; 7 School of Health Professions Education (SHE), Faculty of Health Medicine and Life Sciences (FHML), Maastricht University, Minderbroedersberg 4-6, Maastricht, The Netherlands; 8 Department of Human Genetics, Amsterdam UMC, Vrije Universiteit Amsterdam, De Boelelaan 1117, Amsterdam, The Netherlands; 9 Advisium’s Heeren Loo Zorggroep, Berkenweg 11, Amersfoort, The Netherlands; 10 Association Francophone des Glycogénoses, Guyancourt, France

**Keywords:** Clinical practice guidelines, Patient partnership, Genetic disorder, Intellectual disability, Congenital malformation, Rare disease, Focus groups

## Abstract

**Background:**

Individuals living with rare congenital malformations and/or intellectual disability often face challenges in accessing appropriate healthcare. Clinical practice guidelines (CPGs) may serve as a tool to provide evidence-based care for rare diseases, but their development is complex, and the views of affected individuals and families often remain unknown.

**Methods:**

Patient advocates of the European Reference Network ITHACA (Intellectual disability, TeleHealth, Autism and Congenital Anomalies) participated in focus groups in which their experiences with and perspectives on CPG use and development were discussed.

**Results:**

Patient advocates considered CPGs relevant to address information and care needs and support advocacy efforts. Important characteristics included representation of heterogeneity within conditions, a holistic approach in which and how topics are addressed, user-friendly availability for individuals and families, and reliability of information. Guideline development and implementation were described as challenging, iterative processes in which effective partnership between clinicians, patient advocates, and other stakeholders is essential.

**Conclusions:**

Understanding the perspectives of patient advocates is essential to develop CPGs that meet the life-long and complex care needs of individuals and families living with rare conditions. Identified challenges include balancing the urgency of information needs with thorough guideline development processes, as well as the integration and interpretation of different types of knowledge.

**Supplementary Information:**

The online version contains supplementary material available at 10.1186/s13023-025-03673-9.

## Background

Individuals with rare conditions, such as rare congenital malformations and/or intellectual disability, often face long diagnostic journeys and unmet healthcare needs [1]. Healthcare professionals may lack the knowledge to manage these rare and complex conditions effectively. European Reference Networks (ERNs) have been established as virtual networks of healthcare providers to facilitate collaboration and improve care for rare diseases across Europe [2]. The development and implementation of guidelines are an important pillar of the ERNs to standardize quality of care and treatment, ensuring that it is effective, efficient, and people-centred [1, 2].

Clinical practice guidelines (CPGs) provide evidence-based recommendations to guide clinical decision-making and support healthcare professionals in providing optimal care. CPGs are developed by expert panels through systematic approaches. Recognition of patient involvement in guideline development is increasing, driven by political-ethical demands for patients to be included on a more equal level in health policy; moreover, patient involvement is thought to increase the relevance and applicability of guidelines by drawing attention to aspects that matter in daily life [3].

Currently, there is no standard approach for patient involvement in CPGs [3, 4]. Empirical evidence suggests that patient participation in CPG development does not necessarily result in more patient-centred guidelines [5, 6]. Still, few studies have explored the opinions and experiences of patient advocates concerning guidelines [7, 8], and it is unknown whether these results are transferable to rare disease contexts.

The development of CPGs for rare diseases poses notable challenges, due to a limited evidence base and small numbers of experts. In this context, persons living with rare conditions may contribute valuable insights into the challenges they face and potential solutions of which healthcare professionals might not be aware. As individuals and families often play active roles in orchestrating care in rare disease settings [9, 10], they are also important guideline end-users.

In this qualitative focus group study, experiences with and perspectives on the use and development of CPGs among patient advocates involved with ERN-ITHACA (Intellectual disability, TeleHealth, Autism and Congenital Anomalies) on Rare Congenital Malformations and Rare Intellectual Disability were explored. The study describes (1) the relevance of CPGs to individuals and families living with rare congenital malformations and/or intellectual disability, (2) guideline characteristics that patient advocates consider important, and (3) their experiences and opinions on involvement in the guideline development process.

## Methods

### Study population and recruitment

The study population consists of the rare disease association representatives in the ERN-ITHACA Patient Council in the scope of rare (multiple) malformation syndromes and rare intellectual and other neurodevelopmental disorders of genetic, genomic/chromosomal or environmental origin, both diagnosed and undiagnosed. As appointed European Patient Advocacy Groups (ePAG) advocates, they represent the voices and interests of their patient community as a whole and have a mandate to represent their member organizations within the ERNs. These include organizations for conditions such as the 22q11.2 deletion, AGO2, Angelman, Coffin-Lowry, Cornelia de Lange, CTNNB1, Kleefstra, Noonan, Pitt-Hopkins, Prader-Willi, SATB2-associated, and Williams syndromes, KIF1A-associated neurodegenerative disorder, CDKL5 deficiency disorder, and spina bifida and hydrocephalus, as well as broader rare disease and/or intellectual disability groups. Patient representatives present at the annual ERN-ITHACA Board Meeting were invited to join during the meeting; all Patient Council members were invited through email.

### Data collection

Data was collected through in-person and online focus groups moderated by M.K. and C.G., who both received prior training in interviewing techniques. The focus group guide (Supplementary data) was developed through discussions among the full research team and consisted of broad, open-ended questions to explore experiences and opinions regarding (1) using guidelines and (2) developing guidelines. The order of these two main topics was alternated between group discussions.

In-person focus groups were organized during the annual three-day ERN-ITHACA Board Meeting in Budapest in December 2022, after a brief presentation in which M.K. introduced herself and described ongoing CPG development efforts within the network. Two simultaneous focus groups with five and six participants respectively, lasting approximately 1.5 h, were moderated by M.K. and C.G. in separate rooms at the meeting venue.

Three digital focus groups were organized to allow all ePAGs to participate and to further explore topics that arose in the two in-person discussions. These were conducted through Microsoft Teams in March 2023 with five, two, and three participants respectively, moderated by M.K. and lasting 1–1.5 h. Although we aimed for four or five participants per online group, smaller sessions were also conducted to facilitate all interested patient representatives to participate in accordance with their professional and caregiver schedules.

### Data analysis and reporting

Focus groups were audio recorded and transcribed verbatim; for digital focus groups, video recordings were available. Thematic analysis was conducted inductively. The analytic approach was based on the six-step approach of Braun and Clarke [11]: (1) familiarization with the data through reading and rereading; (2) generating initial, descriptive codes from the data; (3) grouping similar codes into potential themes to identify patterns in the data; (4) reviewing the data to refine the themes as necessary; (5) defining and naming the themes; and (6) presenting the findings by describing the themes, supported by quotations from all focus groups. MAXQDA 2020 software was used to manage and analyse the data [12].

Two researchers, M.K. and C.d.M., who had prior experience in qualitative data analysis, independently coded all focus groups. The initial themes were generated based on the two in-person focus groups. Subsequent digital focus groups were organized and coded iteratively until all interested ePAGs had participated. No new (sub)themes were identified in the last focus group, suggesting inductive thematic saturation [13]. The two researchers discussed the coding of each focus group until consensus was reached; emerging themes were discussed with all co-authors.

Member checking was conducted by sharing a summary of the main themes and subthemes for commentary with all 21 participants through email, to which eleven participants responded. The overall thematic structure was confirmed by all respondents; six provided additional nuances that were integrated into the discussion and phrasing of the manuscript. The results of the focus group study were discussed at the Patient Workshop during the annual ERN-ITHACA Board Meeting in Dublin in December 2023.

Consolidated criteria for reporting qualitative research (COREQ) guided the reporting of the study [14].

### Research team and reflexivity

The study was planned and conducted by an interdisciplinary research team consisting of M.K. (PhD-researcher on guideline development, trained as a medical doctor and care ethicist), A.H. (ERN-ITHACA project manager with lived experience as a parent, > 30 years of active involvement in (inter)national patient advocacy, and experience in > 25 rare disease guideline projects), M.C. (professor of community genetics and public health genomics), C.G. (ERN-ITHACA guideline methodologist with PhD in patient involvement in rare disease clinical trial design), and A.v.E. (ERN-ITHACA Guideline Working Group chair and intellectual disability physician). Data-analysis was conducted inductively by M.K. and C.d.M. (PhD-researcher on guideline development education, without involvement with ERN-ITHACA); during this process, the full research team discussed the themes and interpretations at different stages.

## Results

### Participant characteristics

In total, 21 participants joined the study. Five ePAGs did not participate; three did not have time due to the workload of managing national patient organizations and two did not respond to the invitations. One participant was a local patient representative attending the ERN-ITHACA Board Meeting who formally became an ePAG advocate after participation in the focus groups. Most participants were parents of a child with rare congenital malformations and/or intellectual disability; two participants were affected individuals. Participants came from 16 European countries; some had lived in multiple countries. All participants were active (board) members of associations representing rare diseases, intellectual disability, and/or specific conditions.

### Research findings

**Fig. 1:**
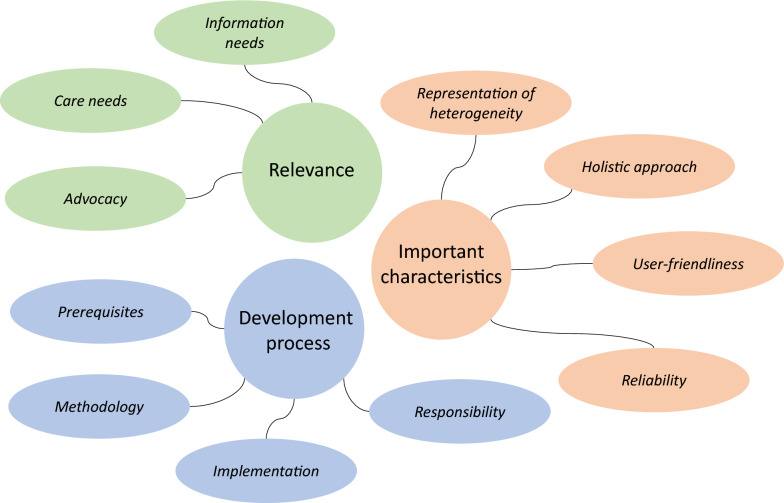
Themes with main subthemes

Below is described (1) why CPGs matter to individuals and families living with rare congenital malformations and/or intellectual disability, followed by (2) which guideline characteristics patient advocates consider important and (3) patient advocate views on the guideline development process (Fig. 1).

#### Relevance of guidelines

**Fig. 2:**
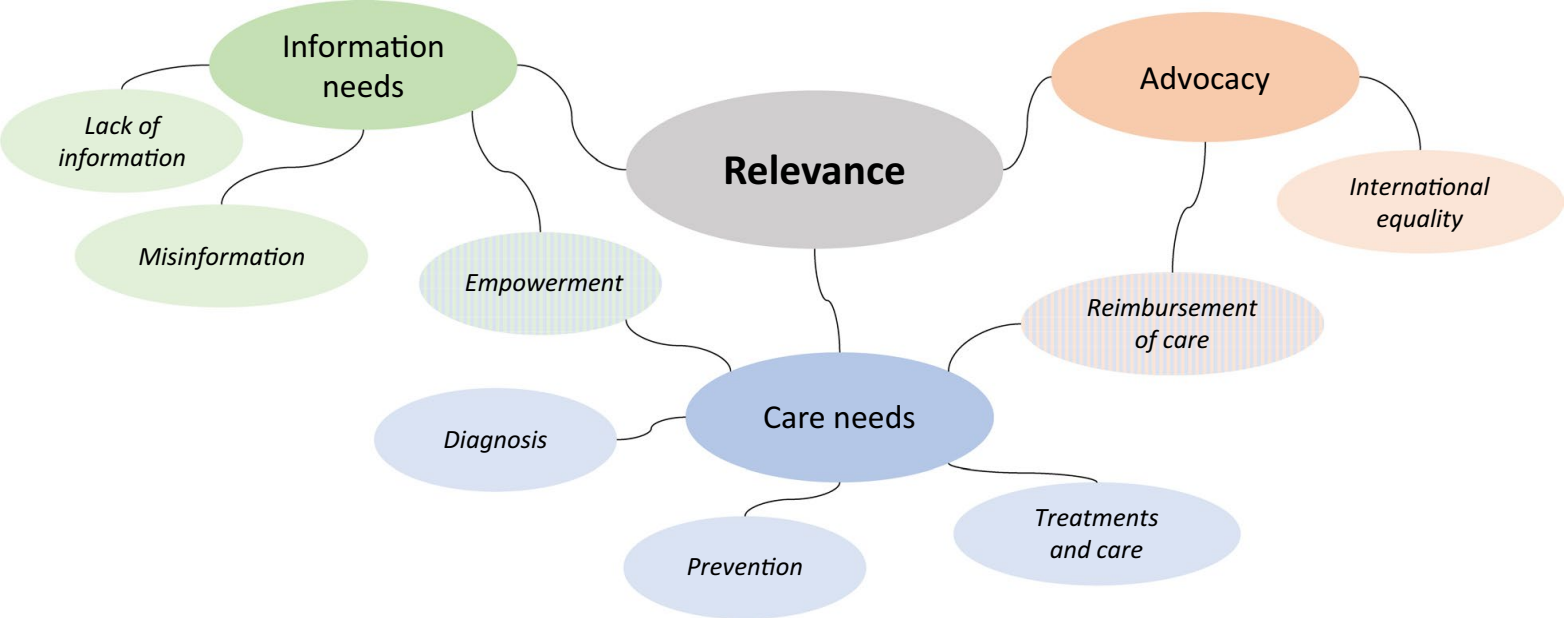
Relevance of clinical practice guidelines for individuals and families with rare conditions

Participants generally deemed guidelines important, describing their relevance with regard to (unmet) information and care needs as well as advocacy efforts (Fig. 2). These needs were strongly intertwined: various participants described a need for information to actively demand care to address their (child’s) needs.

“It is the most common thing, I would say, that patients ask us for, especially those who live (…) in countries, you know, like [in] eastern Europe (…) It is really, how can you get information to those families who are in severe lack of even a specialist doctor” (P20)

##### Information needs

Many participants described that being confronted with having (a child with) a rare condition brings uncertainty: “Parents that have newly diagnosed children are always asking, what should I expect, how is going my child to develop” (P8). Correct and comprehensive information about rare conditions may be difficult to find, and individuals and families often turn to each other in (online) support groups to share experiences. If available, guidelines may constitute a reliable information source. For example, one participant described referring to guidelines to moderate discussions in their support group:

“[In online] support groups, mainly Facebook, (…) different things are being said, and they are not necessarily correct, but for parents, that’s the way it goes, there’s this universe of information and when people hear it enough they think that’s correct. Well, when I am able to say, the guidelines say or the consensus is, you know, it helps.” (P6)

Additionally, guidelines represent a form of ‘expert information’, which is perceived as credible by the outside world. For example, one participant recounted the following interaction with their child’s school: “When I told the teacher not to do certain things, they would not listen, but when I brought some documents from clinicians, oh yes, that is something, so now we have to change” (P5). Guidelines were described as “useful for families to feel empowered” (P10) as they can be used as reference documents in their contacts with healthcare professionals, social workers, and professionals in related fields. In this way, guidelines may support individuals and families in orchestrating care.

##### Care needs

Guidelines were described as helpful to address unmet care needs, including timely diagnosis, adequate treatment and follow-up, and reimbursement of care. For many conditions, there is a long diagnostic journey: “Many people don’t get to know the name of their disease in years (…) We had recently in our syndrome a girl diagnosed with 16 years old” (P12). Participants suggested that guidelines might assist in receiving a diagnosis earlier, through raised awareness of rare diseases and indications for diagnostic testing. Guidelines might also contribute to appropriate treatment and follow-up, for example by setting a standard of care for all healthcare providers: “We are visiting like three different hospitals (…) and each one of them will have [a] different specialist. So if you have like a general guideline, it would be easy for them to follow the same protocols” (P17). Another reported purpose was early identification of and intervention for common comorbidities; for example, one participant explained how increasing scientific knowledge about their syndrome allows for preventative care:

“There is much more prevalence of psychiatric diseases (…) Researchers really did so much study for this syndrome in last twenty years, now we know much more (…) So, you check that, if it is not, okay, but if it is, you can do some things to prevent full-blown psychosis, for example, (…) you can introduce some pharmacotherapy or some psychotherapy.” (P21)

##### Advocacy

Beyond the individual level, participants stated that guidelines support patient advocacy for better healthcare and social policy. Examples included recognition of rare conditions to receive social benefits and reimbursement of (para)medical therapies.

“If we have these guidelines (…) we can pursue more national, legal goals (…) For example, my child needs physiotherapy and speech therapy, one therapy per day, five days per week, then the government knows that they need to fund this amount of hours for this amount of children, but if there is no scientific proof that my child needs that, this need does not exist for the decision makers.” (P8)

Guidelines were also mentioned as a tool for reducing international health disparities, as local patient groups might refer to the standard of care described in (international) guidelines to support their demands for better healthcare: “If you go probably to South America and they see that they are not able to have kind of test, because the system they don’t pay, they have some reason and some evidence what they can look for” (P14).

#### Important characteristics

**Fig. 3:**
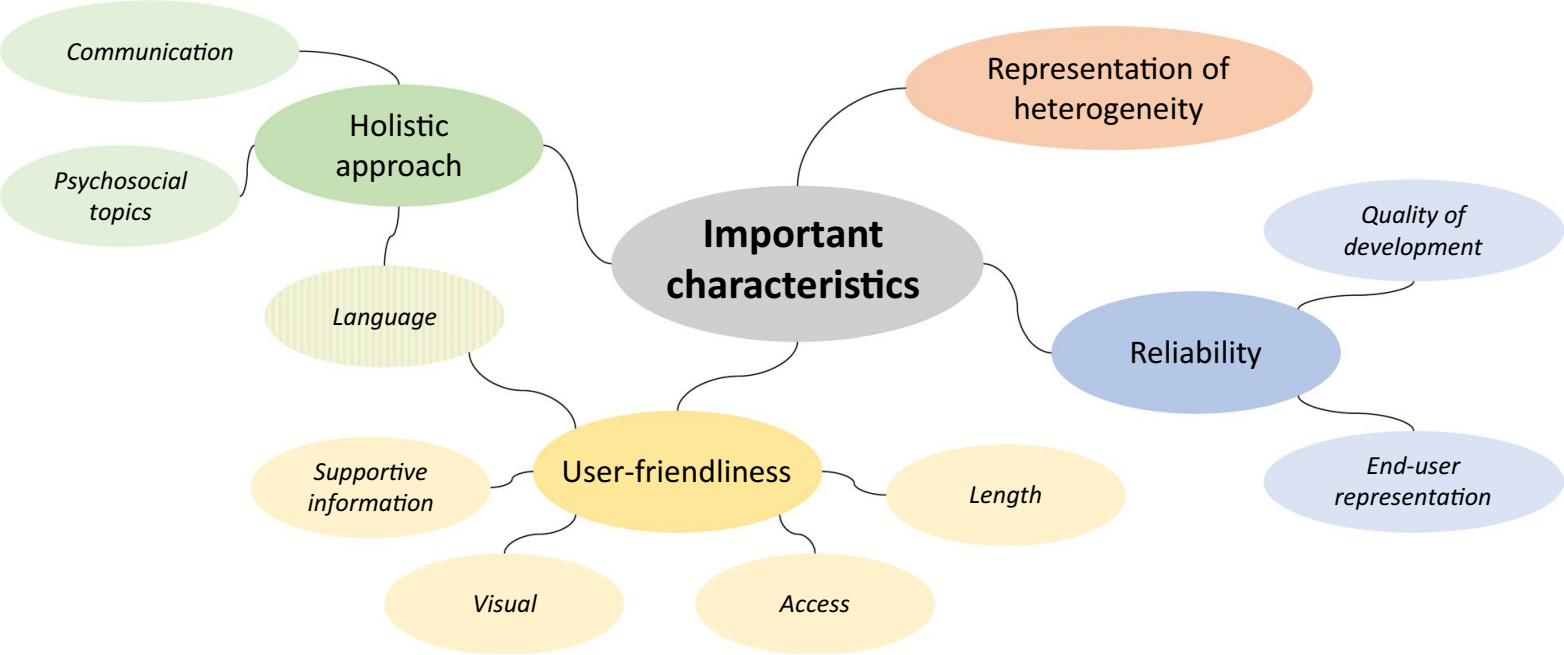
Important characteristics of clinical practice guidelines

Representation of the heterogeneity of individuals, holistic approaches, presenting information in a user-friendly way, and providing reliable information were highlighted as requirements for guidelines to be useful to the patient community (Fig. 3).

##### Representation of heterogeneity

Many participants stressed the importance of acknowledging the heterogeneity of manifestations within individuals with a particular condition in syndrome-specific guidelines. Phenotypic variability is common in genetic neurodevelopmental disorders, meaning that two individuals with the same condition may live different lives and have differing healthcare needs.

“We have mild, moderate and severe cases, (…) probably for all rare neurodevelopmental diseases this is the same, and all of the clinicians have to be aware of [these] unique Prader-Willi, unique Kleefstra, unique Rett patients (…) This exam, maybe it is not going to be applied for this unique person, because he is not experiencing [these] symptoms.” (P1)

Needs may also change throughout life, with different healthcare priorities per age and life phase. Various participants stated concerns about most expertise being centred on care for children, with a lack of knowledge regarding the transition to adult care and ageing:

“When it is a spectrum, it is very difficult to have one specific guideline to cover everything, especially when you have an aging group of people. Now, the paediatric guidelines are not working for me, for example. I need something else. And it is unfortunately not there.” (P16)

##### Holistic approach

Participants highlighted that the life-long nature of these conditions and their varying needs in all areas of life emphasize the importance of a holistic approach: “For our syndrome, that doesn’t have a (…) pharmacological therapy, it is very important to look at the person as a whole and not a piece of them” (P19). In this light, several participants disliked the term ‘patient’, as it refers to individuals based on their medical condition and does not recognize their identity beyond the diagnosis, but rather preferred the use of person-first language.

Living with a rare condition or disability was seen as intertwined with daily life, such that psychosocial topics including education, work, and relationships should be included in guidelines.

“A patient is, as all we are, human. We are very complex, and especially in terms of rare disease and (…) disabilities, non-functioning in some areas like school, work, social (…) relations (…) If the healthcare system is saying, I don’t see all these other worlds, I want to focus in my world because I want to deliver good health for my patient, then the picture is not full.” (P4)

Regarding the content and phrasing of recommendations, several participants referred to a “life-disease balance” (P4) between focusing on health outcomes and enjoying life: “If [my child] has a little ice cream it will be okay, he will not die (…) The quality of life is what matters” (P5). It was also suggested that strongly phrased recommendations could be experienced as stressful by caregivers: “[When it] sounds imperative, it can make some parents very nervous, like am I not doing it right” (P12).

##### User-friendliness

Participants considered individuals and families as active end-users, for whom guidelines must be made accessible and intelligible: “If you have a patient [who isn’t] so into medical language, they can read the lay version, but you should have the extra big one for those that like me want to know everything, every little ittybit” (P15). Shorter summaries, easy-to-read text, and visual and/or video versions were suggested to improve the comprehensibility of guidelines. Free dissemination of both online and printed information was recommended. For international guidelines, translations to national and local languages were mentioned as necessary. Finally, participants reported that guidelines should refer to further supportive information, such as contact information and websites of support groups and centres of expertise for the specific condition.

##### Reliability

Various participants, speaking as leaders of national family organizations, described the responsibility to their community to share reliable information: “We feel responsible, as an association, all the information to be filtered, and of course checked with the doctors, in order to be sure that [it] is creditworthy and deserves to be communicated to the families” (P11). Guideline reliability was seen as a necessity for its use: “The quality is like, initial condition. The best possible or nothing” (P4). Reliability of the guideline, derived from careful development in collaboration with all relevant experts, was also seen as a prerequisite for implementation: “So it reflects expertise of the different groups, that also has a certain validity to it, so that that is adopted” (P3).

#### Development process

**Fig. 4:**
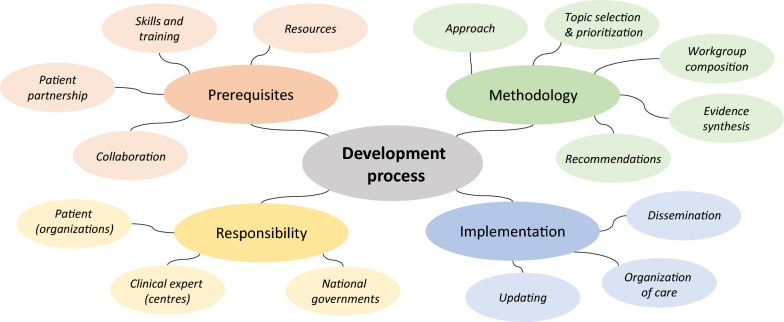
Development process of clinical practice guidelines

The guideline development process was described with regard to its prerequisites, the employed methodology, implementation, and responsibilities for these processes (Fig. 4).

##### Prerequisites

Several prerequisites for successful guideline development were discussed. These factors included collaboration on an international and multidisciplinary level, patient partnership, and having sufficient resources. Participants emphasized that guideline development for rare and complex conditions requires an inclusive approach involving clinical and scientific experts in various fields, affected individuals and families, and governance actors. The participation of patient representatives as equal partners throughout the process was considered essential by all participants. The knowledge of families was seen as complementary to and equally valuable as clinical expertise:

“The perception of parents (…) is critical, but when we talk about pure clinical facts, the experts working with this kind of symptoms (…) have seen many cases, (…), [while] I don’t have experienced further than my child (…) There [are] two parts, the clinical part which are facts that are supported by evidence, and then there is the part of prioritization for the parents (…) to be able to have a manageable family life, and these are two different perspectives.” (P12)

The experiences of participants who had contributed to guideline development varied from being a valued partner in a successful project to being excluded from discussions or struggling to contribute effectively. Guidance and support were thought helpful to allow patient representatives to function optimally:

“I find it difficult to represent the whole community (…) There are very many manifestations and as representatives, we were two family members against (…) 18 or 20 specialists. Specialists all do a literature study for scientific proof. It might be an idea if (…) there would also be an analyst helping the representative analysing, for example, the [patient] stories.” (P10)

##### Methodology

Participants with experience in guideline working groups described development as an iterative process, in which various methodological decisions needed to be made. Discussions included whether to develop syndrome-specific or more generic guidelines; to develop global minimum-standard guidelines or focus on the local level; and how to allocate efforts to guideline development versus clinical research when there is limited evidence. Several participants argued for the development of registries and research agendas to stimulate the generation of further information.

Participants suggested various contributions of affected individuals and families, including prioritization of topics that are important in everyday life, sharing information on (underreported) comorbidities and current care, and assessing the impact of recommendations on the patient experience. Both active collection of perspectives through surveys, focus groups, or patient stories, and the knowledge patient representatives implicitly derive from their work and daily life, were mentioned.

“As parents, we can fill the gaps that currently exist on the odyssey (…) of our rare disease (…) We can see, when we visit to the neurologist, there is a problem with the EEGs because they are not performed (…) so we can fill those gaps, and the rest can be [written] down by professionals, but we can say, hey, there is a gap here, there is a gap there.” (P18)

Considering the large number of rare conditions and the scarcity of existing guidelines, efficiency of development was seen as important: “Maybe we need new methodology to approve guidelines, in order for this generation to have a guideline, because somehow it takes so long” (P9). In general, participants described a sense of urgency to provide information, which might conflict with the desire to make a reliable guideline:

“It takes us one year for making this [document], to read all the information, to ensure it is correct (…) so each time it is a discussion (…), it is important, and in the background you have the families asking, when, when, when, and I say, I prefer waiting maybe one, two, three months more to have a good information. If I give you something that is not correct, it is also my responsibility as a president of the organization.” (P7)

##### Implementation

Participants considered guideline implementation as essential for individuals living with a rare condition to benefit from its development. This was seen as neither automatic nor straightforward:

“The guidelines are just an idea of what we have to do. Then, we have to write who is the leader (…), who are the other actors who are involved in the pathway, and which hospitals or local units have to do that, in which time, when, and so on. So, there is a guideline, and then there is a translation on who, when, how.” (P19)

Dissemination to both healthcare providers and affected individuals and families was perceived as crucial. Various participants described that they or their community members tend to receive care from healthcare providers with limited knowledge of their condition. Contacts with healthcare and government actors were reported to share information and raise awareness: “The first step [is] to inform doctors” (P2). On the other hand, dissemination of the guideline to affected individuals and families was highlighted as important, enabling them to actively demand care: “It is the parents that are very often going to be the persons to take the guideline along. If they feel more part of them, (…) it is not beyond them to take them” (P6).

Other mentioned challenges included differences in health systems between countries, limited funding and expertise on the local level, and the lack of strategies to update guidelines after initial development. Several participants stated that these challenges need to be discussed early on:

“How much in detail do you go, do you write very general and then you don’t add much to the already existing (…) situation, or if you go in too much into details, let’s say, (…) this examination must be done every three years or so, then you have a problem on the local level because the insurance doesn’t cover this examination.” (P13)

##### Responsibility

Participants described an active role for patient associations across the guideline development and implementation process. Through their networks, these organizations may provide access to the experiences and opinions of individuals and families. Subsequently, the associations may play a role in adapting guidelines to local settings and promoting dissemination and implementation among national actors.

“That is the importance of the association, because (…) we are the engine for this work (…) We are connecting people, connecting clinicians, putting them together to talk about the disease.” (P19)

Some participants reported that the responsibility for guidelines should be shared across all stakeholders who are part of the guideline development process. For example, clinical experts and governance actors have access to different mechanisms and resources to promote implementation.

“A lot of centres only (…) start working with (…) guidelines if they are accredited (…) Once [accreditation] is in place, that means that you already have stakeholders there on a national level and on centre level. And then you have the patients who can actually furthermore advocate it, and then, top-down and bottom-up, you have two approaches that can work” (P3)

## Discussion

In this focus group study among European patient advocates for rare congenital malformations and/or intellectual disability, CPGs were considered important tools for information provision, improving care, and advocacy on national and international levels. Guideline development and implementation for this population were described as iterative processes that require collaboration between all stakeholders, including the involvement of patient representatives as equal partners. It is important to recognize patient advocates’ aims and priorities for guideline development, as their perspectives may differ from those of other stakeholders, and to ensure patient partnership throughout the development process, for which recommendations are provided in Table 1.

**Table 1:** Recommendations for patient partnership by guideline development stage

Overall	Collaborate with patient representatives from the initial stages; Facilitate partnership through training, guidance, and/or support where necessary
Prioritization	Prioritize topics that are relevant to individuals and families living with rare conditions
Preparation	Create multidisciplinary guideline working groups in which patient representative input is facilitated and acknowledged
Data synthesis	Collect information from patient communities through quantitative (surveys, patient-led registries) and qualitative methods (focus groups, interviews, patient stories)
Formulation of recommendations	Include patient views in considerations to inform the content and phrasing of recommendations
Implementation	Collaborate with (national) patient organizations in guideline dissemination and implementation

### Balancing reliability and urgency to address unmet needs

This study identified a tension between reliability through rigorous methodology, associated with lengthy development processes, and a sense of urgency to make a wide range of information available as soon as possible. This contradiction may result from the multiple responsibilities patient advocates hold to address the unmet information and care needs of their communities. Guidelines were described as a tool to empower affected individuals and families in accessing appropriate healthcare and social support, in line with the role of the patient as an active partner in rare disease management. [9, 10] Although the burden placed on individuals and caregivers should not be overlooked, explicitly recognizing them as end-users means dissemination and user-friendliness for this audience are essential. A recent review of the information needs of caregivers of individuals with rare epilepsy syndromes and intellectual disability showed a diverse range of needs, from diagnosis-specific medical information to support in navigating health system organization and coping strategies. [15] In this light, it is important to assess which information and care needs are most pressing and to determine whether guideline development or other policy efforts are the best way to meet those needs.

### Navigating diverse goals

The various goals patient advocates aim to achieve using guidelines may require different development strategies: for example, strongly phrased minimum-standard CPGs may be powerful tools to support advocacy, but may leave less room for sharing daily life experiences and freedom of choice in terms of the “life-disease balance”. Patient advocate goals can also differ from those of other stakeholders: where guideline methodologists and clinical experts may aim to provide general recommendations for clinical management, participants highlighted the importance of personalized and holistic approaches to unique individuals, including psychosocial topics. Heterogeneity is a known challenge in rare disease research and may similarly complicate the formulation of clinical practice recommendations that apply to all individuals with a particular condition [16]. An existing framework that could help to comprehensively address all life domains is the International Classification of Functioning and Disability (ICF) [17, 18].

### Harmonizing knowledge paradigms

Guideline development may reveal epistemic conflicts, as participants consider information from individual lived experiences to be highly valuable, while CPGs rely strongly on population-level evidence from scientific research [5, 19]. Ethnographic research of guideline development panels has described the difficulties of integrating different types of knowledge in CPGs, in which approaches focused on high-quality clinical evidence (e.g. defined as meta-analysis of randomized controlled trials) may conflict with more pragmatic understandings of relevant knowledge including clinical expertise, patient experiences, and real-world data [20]. As the perceived validity of CPGs is essential to the aims participants describe, including convincing healthcare professionals and policymakers and providing reliable information to families, the question as to which knowledge is considered reliable holds particular importance.

### Facilitating patient partnership

All participants consider patient partnership as a prerequisite for effective guideline development. Suggestions for collaboration at each stage of the development process are provided in Table 1. This continuous and broad conception of partnership confirms and extends upon previous research and policy reports that recommend combining consultation, deliberation, and co-decision methods throughout all stages of guideline development [21–23]. It is important to note that not every condition or country has a patient organization and that skills and experiences might vary between representatives. Training on guideline methodology and terminology, guidance and support during the development process, and/or (financial) compensation for and practical consideration of the commitment required of patient advocates could help secure patient representation in guideline development [6, 21].

### Strengths and limitations

By speaking to all interested members of the ERN-ITHACA Patient Council, who are both mandated to speak on behalf of their community and experienced in discussing policy issues, we believe our study holds high information power [24]. To accommodate participation of this international group, digital focus groups were a necessity; previous research supports the use of virtual focus group formats in rare disease contexts [25]. Most participants were parents of an affected child; all were officially mandated by their patient organizations, have professional working proficiency in English, and some have a professional or educational background in healthcare or research. Although these individuals represent larger patient communities through their organizations, their experiences may not reflect those of all individuals and families living with rare congenital malformations and/or intellectual disability. An important limitation is that no individuals with intellectual disability participated in the focus groups, and this work might not reflect their perspectives. The topic of patient representatives speaking as proxies on behalf of affected individuals was only briefly mentioned during the focus groups. Collaboration with individuals with intellectual disability in healthcare research and guideline development receives increasing attention [26] and needs to be considered in future efforts. Within a multiple-strategy approach to patient partnership, collaboration with patient organization leaders, families and caregivers, and affected individuals can coexist.

## Conclusion

In this focus group study, patient advocate perspectives on guideline development for rare congenital malformations and/or intellectual disability were explored. Patient advocates viewed CPGs as a tool to meet information and care needs and support their advocacy work. Representation of heterogeneity, holistic approaches, user-friendly dissemination, and reliable development were considered important. The study identified tensions between evidence-based methodologies and the value placed on experiential knowledge as well as the speed of guideline development. The goals various stakeholders aim to achieve through guideline development deserve further attention in research and policy efforts. Supporting patient partnership is recommended to improve the relevance and implementation of guidelines.

## Supplementary Information

Additional file 1.

## Data Availability

The data generated during the current study are not publicly available due data containing information that can be traced back to individuals, but are available from the corresponding author in consultation with the ERN-ITHACA Patient Advisory Board on reasonable request.
