# Supplementary material for: ‘We are the engine’: a focus group study on clinical practice guideline development with European patient advocates for rare congenital malformations and/or intellectual disability
Source: Orphanet J Rare Dis. 2025 Apr 10;20:169. doi: 10.1186/s13023-025-03673-9 (PMC11983842; doi:10.1186/s13023-025-03673-9)
Supplement: Supplementary file 1 — Additional file 1. [file 13023_2025_3673_MOESM1_ESM.docx]

Supplementary data to *‘We are the engine’: A focus group study on clinical practice guideline development with European patient advocates perspectives on clinical practice guideline development for rare congenital malformations and/or intellectual disability.*
 **Focus group guide**

- Introduction (purpose and planning of the focus group, participants)
  - Introducing moderator; purpose (“*We are interested in hearing your opinions on the use and the development of guidelines*”); planning (duration); conversation rules (everyone’s opinion is important; telephone on silent mode); recording for scientific article will be saved at Amsterdam UMC and deleted after 1 year; start recording.
- Introductory round: name and your role in ERN-ITHACA/PO.
- **Topic 1*:** Experiences in using guidelines within patient organization (PO) and needs regarding guideline end-products
  - Introductory round**:** short round to ask who has ever used a guideline.

    *Possible questions*:
  - Do(es) you/your PO have experience with using guidelines or guideline tools, such as decision aids?
  - What is important to you/your PO to use guidelines or guideline tools?
- **Topic 2*:** Experiences in participating in guideline development as part of patient organization/patient representative (PR)
  - Introductory round**:** short round to ask who has previously participated in guideline.

    *Possible questions*:
  - Do(es) you/your PO have experience with participation in the development of guidelines? Could you tell me about these experiences?
  - What is your opinion on participation of PR in guideline development?
  - In which phase(s) of guideline development should PR participate?
  - Through which methods should PR be involved in guideline development?
- Rounding up: any points that have not been mentioned yet?
- Closing: thanking participants and stopping recording

** The order of these two main topics was alternated between group discussions.*
